# Predictors for Stroke and Death in Non-Anticoagulated Asian Patients with Atrial Fibrillation: The Fushimi AF Registry

**DOI:** 10.1371/journal.pone.0142394

**Published:** 2015-11-05

**Authors:** Yasuhiro Hamatani, Yugo Yamashita, Masahiro Esato, Yeong-Hwa Chun, Hikari Tsuji, Hiromichi Wada, Koji Hasegawa, Mitsuru Abe, Gregory Y. H. Lip, Masaharu Akao

**Affiliations:** 1 Department of Cardiology, National Hospital Organization Kyoto Medical Center, Kyoto, Japan; 2 Department of Arrhythmia, Ijinkai Takeda General Hospital, Kyoto, Japan; 3 Tsuji Clinic, Kyoto, Japan; 4 Division of Translational Research, National Hospital Organization Kyoto Medical Center, Kyoto, Japan; 5 University of Birmingham Centre for Cardiovascular Sciences, City Hospital, Birmingham, United Kingdom; 6 Aalborg Thrombosis Research Unit, Department of Clinical Medicine, Aalborg University, Aalborg, Denmark; Indiana University, UNITED STATES

## Abstract

**Background:**

Atrial fibrillation (AF) increases the risk of stroke and death. Data on the predictors for stroke and death in ‘real-world’ AF patients are limited, especially from large prospective Asian cohorts.

**Methods:**

The Fushimi AF Registry is a community-based prospective survey designed to enroll all AF patients who visited the participating medical institutions in Fushimi-ku, Kyoto, Japan. Follow-up data were available for 3,304 patients (median follow-up period 741 days). We explored the predictors for ‘death, stroke, and systemic embolism (SE)’ during follow-up in 1,541 patients not receiving oral anticoagulants (OAC) at baseline.

**Results:**

The mean age was 73.1 ± 12.5 years, and 673 (44%) patients were female. The mean CHADS_2_ and CHA_2_DS_2_-VASc scores were 1.76 and 3.08, respectively. Cumulative events were as follows: stroke/SE in 61 (4%) and death in 230 (15%), respectively. On multivariate analysis, advanced age (hazard ratio (HR): 1.68, 95% confidence interval (CI): 1.24–2.29), underweight (body mass index <18.5 kg/m^2^) (HR: 1.71, 95% CI: 1.25–2.32), previous stroke/SE/transient ischemic attack (HR: 1.70, 95% CI: 1.25–2.30), heart failure (HR: 1.59, 95% CI: 1.17–2.15), chronic kidney disease (HR: 1.53, 95% CI: 1.16–2.02), and anemia (HR: 2.41, 95% CI: 1.78–3.28) were independent predictors for death/stroke/SE. Cumulative numbers of these 6 risk predictors could stratify the incidence of death/stroke/SE in patients without OAC, as well as those with OAC in our registry.

**Conclusions:**

Advanced age, underweight, previous stroke/SE/transient ischemic attack, heart failure, chronic kidney disease, and anemia were independently associated with the risk of death/stroke/SE in non-anticoagulated Japanese AF patients.

## Introduction

Atrial fibrillation (AF) is a common cardiac arrhythmia among the elderly [1], and is a well-established risk factor for stroke/systemic embolism (SE) and death [2, 3]. While many large-scale randomized clinical trials (RCTs) of AF patients were conducted to investigate the efficacy and safety of non-vitamin K antagonist anticoagulants for stroke prevention, they do not necessarily represent patients routinely seen in clinical practice due to trial-specific inclusion/exclusion criteria. Indeed, the original historical RCTs of stroke prevention only randomized <10% of patients screened, and many stroke risk factors were not recorded nor consistently defined [4]. Thus, RCTs should be complemented by ‘real-world’ registries. To date, there have been many reports about risk factors for stroke/SE in patients with AF, but most have been based on the data from RCTs or registries with some inclusion/exclusion criteria, with most large prospective studies being derived from Western countries. Given the global burden of AF, it is important to have ‘real-world’ data on the clinical epidemiology of this common arrhythmia from Asian countries.

Unlike RCTs which are tightly-controlled studies with pre-specified protocols and close follow-up, some deaths could be due to undiagnosed stroke, given the nature of registry studies. Therefore, we defined the primary endpoint as composite of *‘all-cause death*, *stroke*, *and SE (death/stroke/SE)’* in the present study. We aimed to test the performance of CHADS_2_ [5] and CHA_2_DS_2_-VASc scores [6], which are well-validated risk stratification schemes for stroke/SE, for the prediction of the composite endpoint, and second, to derive the potential risk factor predictors for the composite endpoint, using a large-scale prospective ‘real-world’ registry of Japanese AF patients.

## Methods

The Fushimi AF Registry is a community-based prospective survey of AF patients in Fushimi-ku, Kyoto, Japan [7, 8]. The detailed study design, patient enrollment, participating institution, the definition of the co-morbidities or the measurements, and subjects’ baseline clinical characteristics of the Fushimi AF Registry were previously described (UMIN Clinical Trials Registry: UMIN000005834) [7]. The inclusion criterion for the registry is the documentation of AF on a 12-lead electrocardiogram or Holter monitoring at any time. There are no exclusion criteria for this ‘all-comers’ registry. A total of 79 institutions, all of which are members of Fushimi-Ishikai (Fushimi Medical Association), participated in the registry. The participating institutions comprised 2 cardiovascular centers (National Hospital Organization Kyoto Medical Center and Ijinkai Takeda Hospital), 9 small- and medium-sized hospitals, and 68 primary care clinics. The enrollment of patients was started in March 2011. All of the participating institutions attempted to enroll all consecutive patients with AF under regular outpatient care or hospital admission.

Among the registry participants, we analyzed the patients whose follow-up data were available. We defined the primary endpoint as composite endpoint of ‘death/stroke/SE’ during follow-up period. Stroke was defined as the sudden onset of a focal neurologic deficit in a location consistent with the territory of a major cerebral artery, and it was confirmed by computed tomography or magnetic resonance imaging. SE was defined as an acute vascular occlusion of an extremity or organ.

We defined advanced age as more than or equal to 75 years, underweight as body mass index (BMI) at baseline less than 18.5 kg/m^2^ according to the World Health Organization criteria [9], and anemia as value of hemoglobin at baseline less than 13 g/dl (male), less than 12 g/dl (female) according to the World Health Organization criteria [10]. Oral anticoagulants (OAC) included warfarin, dabigatran, rivaroxaban, apixaban, and edoxaban.

### Statistical analysis

Continuous variables are expressed as mean ± standard deviation (SD), or median and interquartile range. Categorical variables are presented as numbers and percentages. We compared categorical variables using the chi-square test when appropriate; otherwise, we used Fisher’s exact test. We compared continuous variables using Student’s t-test on the basis of the distribution. We stratified the entire cohort by OAC prescription at baseline, and compared the baseline characteristics between patients without OAC and those with it.

To investigate the predictors for death/stroke/SE, we analyzed the patients without OAC at baseline. First, we tested the predictive ability of the CHADS_2_ and CHA_2_DS_2_-VASc scores on the composite endpoint of ‘death/stroke/SE’ in patients without OAC, using a receiver-operator characteristic curve analysis (as a measure of the C-index). Thereafter, we performed univariate and multivariate analysis using Cox proportional hazard models to explore the predictors for composite endpoint of ‘death/stroke/SE’. All clinically relevant potential risk factors for death/stroke/SE were included on multivariate analysis. The patients with at least 1 missing covariate were excluded on multivariate analysis. We also analyzed the patients without OAC throughout follow-up period.

We investigated whether the cumulative number of each risk factors which were significant on multivariate analysis could stratify the incidence of ‘death/stroke/SE’ and ‘stroke/SE’ during the follow-up period. The continuous distribution of cumulative risk factors was then stratified into 5 categories, and the cumulative incidence of death/stroke/SE across these 5 categories were estimated by Kaplan-Meier method, and compared by the log-rank test. We also investigated whether the cumulative number of risk factors could stratify the incidence of events in AF patients taking OAC. Finally, we evaluated the model predictive ability for the cumulative number of risk factors on the composite endpoint of ‘death/stroke/SE’ in patients without OAC and in those with OAC, again using a receiver-operator characteristic curve analysis. We used JMP version 10 (SAS Institute, Cary, NC) to perform all analyses. Two-sided P values less than 0.05 were considered as statistically significant.

### Ethics

The study protocol conforms to the ethical guidelines of the 1975 Declaration of Helsinki, and was approved by the ethical committees of the National Hospital Organization Kyoto Medical Center and Ijinkai Takeda General Hospital. Since the present research involves an observational study not using human biological specimens, written informed consent was not obtained from each patient for their clinical records to be used in this study, according to the ethical guidelines for epidemiological research issued by the Ministry of Education, Culture, Sports, Science and Technology and the Ministry of Health, Labour and Welfare, Japan. However, we have published all relevant details regarding this study to be carried out and provide each patient an opportunity to refuse inclusion in this research by posting the details at every participating clinic and at the homepages of our institutions. We also held a public meeting with citizens in Fushimi-ku to demonstrate outlines of the present study. As consent was not obtained, patient records and information was anonymized and de-identified prior to analysis.

## Results

A total of 4,115 patients were enrolled by the end of July 2014. Of 3,666 patients who were enrolled one year before (by the end of July 2013), follow-up data (collected every year) were available for 3,304 patients (follow-up rate: 90.1%). Among 3,304 patients, 1,541 patients were not prescribed OAC at baseline, 1,751 patients were prescribed OAC at baseline, and 12 patients’ prescription data were not available. Median follow-up periods were 736 days (interquartile range: 380 days to 1096 days) in patients without OAC, and 748 days (interquartile range: 458 days to 1112 days) in patients with OAC, respectively.

**Fig 1:**
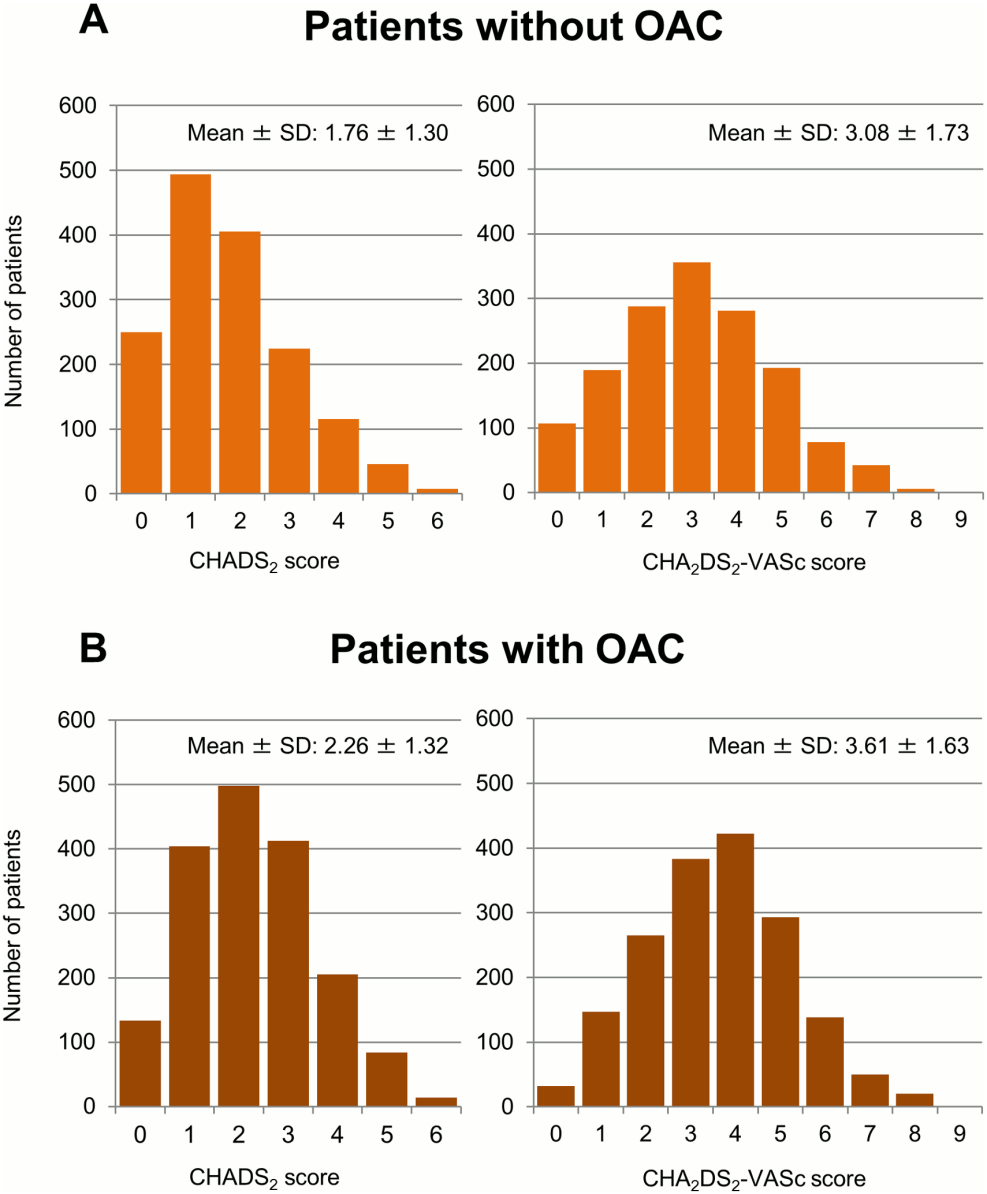
Distribution of patients for each CHADS_2_ and CHA_2_DS_2_-VASc score. (A) Patients without oral anticoagulant (OAC). (B) Patients with OAC. SD: standard deviation.

Baseline characteristics, co-morbidities in patients with and without OAC at baseline are shown in Table 1. Patients without OAC were more often female (p<0.01), younger (p = 0.01), and had lower BMI (p<0.01). Previous stroke/SE/transient ischemic attack (TIA), heart failure, hypertension and diabetes mellitus were less common in patients without OAC (all p<0.01), whereas paroxysmal AF and antiplatelet therapy at baseline were more common in patients without OAC (both p<0.01). The mean CHADS_2_ score and CHA_2_DS_2_-VASc score were lower in patients without OAC (both p<0.01) (Fig 1).

**Table 1:** Comparison of baseline characteristics and co-morbidities between patients with and without OAC at baseline.

	Without OAC	With OAC	p value
Number	N = 1,541	N = 1,751	
<Baseline characteristics>			
Female sex	673 (44%)	651 (37%)	<0.01
Age (years)	73.1 ± 12.5	74.1 ± 9.2	0.01
Advanced age (≥75 years)	759 (49%)	929 (53%)	0.03
Body weight (kg)	57.9 ± 13.2	60.1 ± 13.3	<0.01
BMI (kg/m^2^)	22.7 ± 4.0	23.3 ± 4.0	<0.01
Underweight (BMI <18.5 kg/m^2^)	180 (14%)	138 (9%)	<0.01
Systolic blood pressure (mmHg)	126.3 ± 19.3	123.2 ± 18.6	<0.01
Diastolic blood pressure (mmHg)	70.9 ± 13.0	70.2 ± 12.7	0.10
Heart rate (beats/min)	78.1 ± 16.0	77.4 ± 15.6	0.17
Type of atrial fibrillation			
paroxysmal	963 (63%)	618 (35%)	
Persistent	111 (7%)	148 (9%)	<0.01
Permanent	467 (30%)	985 (56%)	
Antiplatelet therapy at baseline	535 (35%)	433 (25%)	<0.01
<Co-morbidities>			
Previous stroke/SE/TIA	218 (14%)	466 (27%)	<0.01
Heart failure	298 (19%)	580 (33%)	<0.01
Hypertension	905 (59%)	1,116 (64%)	<0.01
Diabetes mellitus	322 (21%)	442 (25%)	<0.01
Dyslipidemia	641 (42%)	785 (45%)	0.06
Coronary artery disease	236 (15%)	251 (14%)	0.43
Valvular heart disease	202 (13%)	389 (22%)	<0.01
Cardiomyopathy	27 (2%)	69 (4%)	<0.01
Peripheral artery disease	58 (4%)	80 (5%)	0.25
Chronic kidney disease	479 (31%)	661 (38%)	<0.01
Anemia	552 (39%)	582 (35%)	0.07
COPD	67 (4%)	95 (5%)	0.15
History of major bleeding	44 (3%)	36 (2%)	0.14

Categorial data are presented as number (%). Continuous data are presented as mean ± standard deviation. OAC: oral anticoagulants, BMI: body mass index, SE: systemic embolism, TIA: transient ischemic attack, COPD: chronic obstructive pulmonary disease.

The rates of major clinical events in patients without OAC during follow-up period were as follows: death/stroke/SE in 270 (9.1 per 100 person-years), all-cause death in 230 (7.7 per 100 person-years), non-cardiac death in 205 (6.8 per 100 person-years), and stroke/SE in 61 (2.1 per 100 person-years), respectively (Table 2).

**Table 2:** Cumulative events and incidence of events during follow-up in patients without OAC at baseline (N = 1,541).

	Cumulative events Number (%)	Incidence of events (/100 person-years)
Death/stroke/SE	270 (18%)	9.1
All-cause death	230 (15%)	7.7
Cardiac death	25 (2%)	0.9
Non-cardiac death	205 (13%)	6.8
Stroke/SE	61 (4%)	2.1
Stroke	59 (4%)	2.0
Ischemic stroke	42 (3%)	1.4
Hemorrhagic stroke	17 (1%)	0.6
SE	3 (0.2%)	0.1

OAC: oral anticoagulant, SE: systemic embolism.

Predictive abilities of the CHADS_2_ and CHA_2_DS_2_-VASc scores on the composite endpoints of ‘death/stroke/SE’ in patients without OAC were modest, with C-indexes of the CHADS_2_ and CHA_2_DS_2_-VASc scores being 0.67 and 0.67, respectively.

On univariate analysis, following variables were significantly associated with increased risk of death/stroke/SE: advanced age (≥75 years), underweight (BMI <18.5 kg/m^2^), previous stroke/SE/TIA, heart failure, valvular heart disease, peripheral artery disease, chronic kidney disease (CKD), and anemia. Variables associated with decreased risk of death/stroke/SE were as follows: paroxysmal AF, and dyslipidemia.

Among 1,541 patients without OAC, 233 patients’ BMI data and 111 patients’ laboratory data of hemoglobin were missing. Therefore, we included 1,245 patients without OAC in a multivariate analysis. Advanced age (hazard ratio (HR): 1.68, 95% confidence interval (CI): 1.24–2.29), underweight (HR: 1.71, 95% CI: 1.25–2.32), previous stroke/SE/TIA (HR: 1.70, 95% CI: 1.25–2.30), heart failure (HR: 1.59, 95% CI: 1.17–2.15), CKD (HR: 1.53, 95% CI: 1.16–2.02), and anemia (HR: 2.41, 95% CI: 1.78–3.28) were independent predictors for death/stroke/SE on multivariate analysis (Table 3). We did not investigate the impact of smoking habit or history of malignancy on death/stroke/SE, due to missing data (the number of patients with unknown smoking habit was 507, and we did not collect the status of malignancy).

**Table 3:** Predictors for the incidence of death/stroke/SE in patients without OAC; Univariate and multivariate analysis.

	Univariate analysis		Multivariate analysis	
Variable	HR (95% CI)	p value	HR (95% CI)	p value
<Baseline characteristics>				
Female sex	1.12 (0.88–1.43)	0.34	0.81 (0.62–1.06)	0.13
Advanced age (≥75 years)	2.93 (2.25–3.84)	<0.01	1.68 (1.24–2.29)	<0.01
Underweight (BMI <18.5 kg/m^2^)	2.76 (2.08–3.64)	<0.01	1.71 (1.25–2.32)	<0.01
Paroxysmal atrial fibrillation	0.59 (0.46–0.75)	<0.01	0.84 (0.64–1.11)	0.22
Antiplatelet therapy at baseline	1.09 (0.85–1.39)	0.50	0.83 (0.62–1.09)	0.18
<Co-morbidities>				
Previous stroke/SE/TIA	2.41 (1.83–3.14)	<0.01	1.70 (1.25–2.30)	<0.01
Heart failure	3.05 (2.38–3.89)	<0.01	1.59 (1.17–2.15)	<0.01
Hypertension	0.88 (0.70–1.13)	0.32	0.81 (0.62–1.05)	0.11
Diabetes mellitus	1.18 (0.89–1.55)	0.24	0.99 (0.72–1.34)	0.96
Dyslipidemia	0.69 (0.53–0.88)	<0.01	0.90 (0.68–1.19)	0.48
Coronary artery disease	1.23 (0.90–1.66)	0.19	0.89 (0.62–1.27)	0.54
Valvular heart disease	2.04 (1.51–2.71)	<0.01	1.05 (0.75–1.46)	0.76
Cardiomyopathy	0.38 (0.06–1.19)	0.11	0.37 (0.06–1.17)	0.10
Peripheral artery disease	1.95 (1.17–3.05)	0.01	1.38 (0.80–2.23)	0.24
Chronic kidney disease	2.70 (2.13–3.43)	<0.01	1.53 (1.16–2.02)	<0.01
Anemia	3.93 (3.04–5.12)	<0.01	2.41 (1.78–3.28)	<0.01
COPD	1.44 (0.83–2.30)	0.18	1.06 (0.60–1.75)	0.83
History of major bleeding	1.62 (0.83–2.82)	0.15	0.84 (0.41–1.52)	0.58

SE: systemic embolism, HR: hazard ratio, CI: confidence interval, BMI: body mass index, TIA: transient ischemic attack, COPD: chronic obstructive pulmonary disease.

In patients without OAC at baseline (n = 1,541), 290 patients (18.8%) started taking OAC during follow-up period. Of patients with OAC at baseline (n = 1,751), 353 patients (20.2%) stopped taking OAC during follow-up. In a sensitivity analysis, we separately analyzed the subgroup of patients who had never taken any OAC throughout the follow-up period (n = 1,251), and our result were consistent, with the following 6 factors (advanced age, underweight, previous stroke/SE/TIA, heart failure, CKD, and anemia) still being independently associated with the incidence of death/stroke/SE on multivariate analysis.

**Fig 2:**
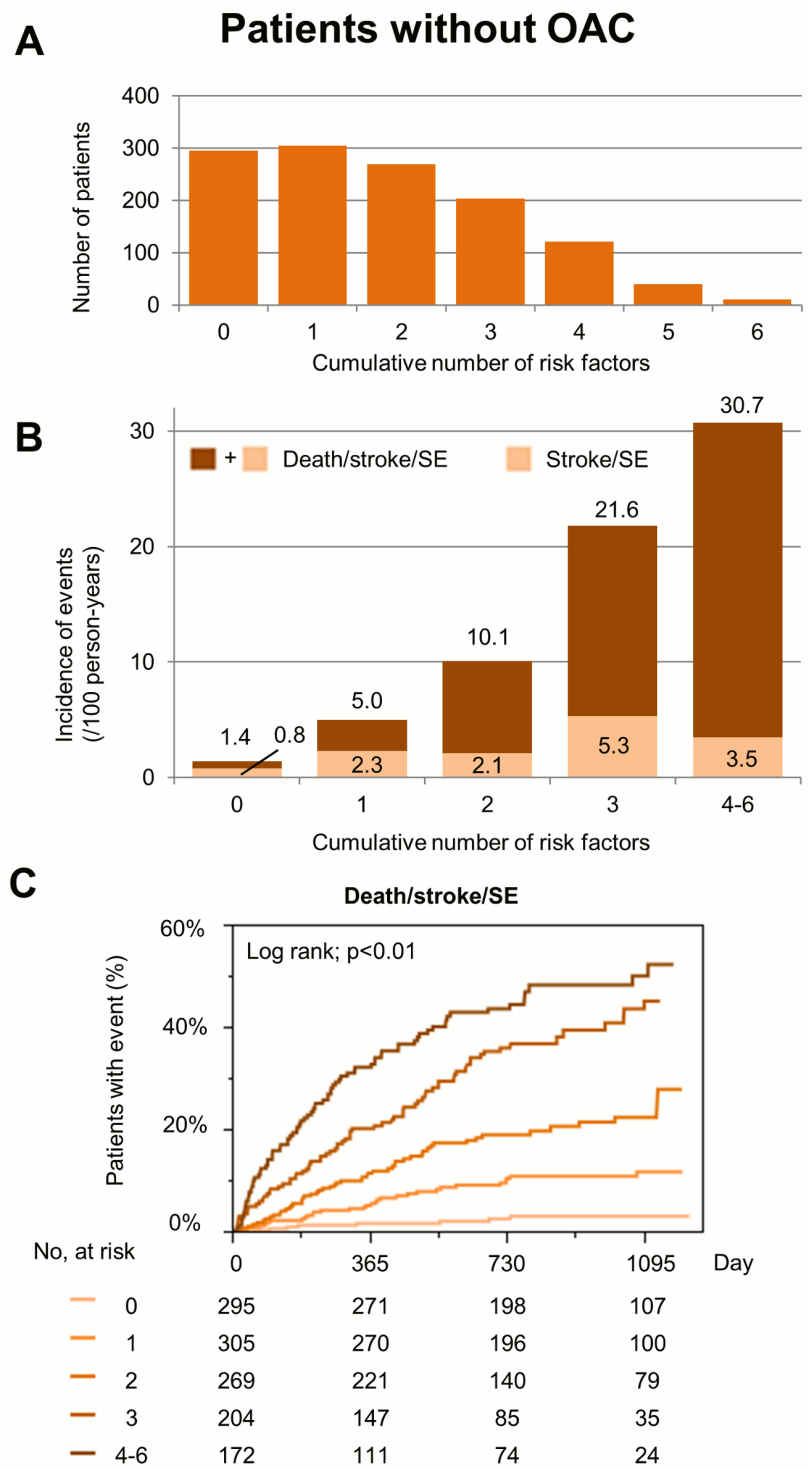
(A) The number of patients for each cumulative number of risk factors in patients without oral anticoagulant (OAC). (B) Incidence of death/stroke/systemic embolism (SE) during follow-up for each cumulative number of risk factors in patients without OAC. (C) Kaplan-Meier curves for the incidence of events during follow-up in patients without OAC. Risk factors are the following 6 components; advanced age, underweight, previous stroke/SE/TIA, heart failure, CKD, and anemia.

Among the patients without OAC, the numbers of patients and the incidence of death/stroke/SE for each cumulative number of 6 risk factors (advanced age, underweight, previous stroke/SE/TIA, heart failure, CKD, and anemia) are shown in Fig 2A and 2B. Kaplan-Meier curves for the incidence of death/stroke/SE during follow-up between risk categories in patients without OAC are shown in Fig 2C. The cumulative number of these 6 risk factors show a significant gradient for the incidence of these endpoints.

**Fig 3:**
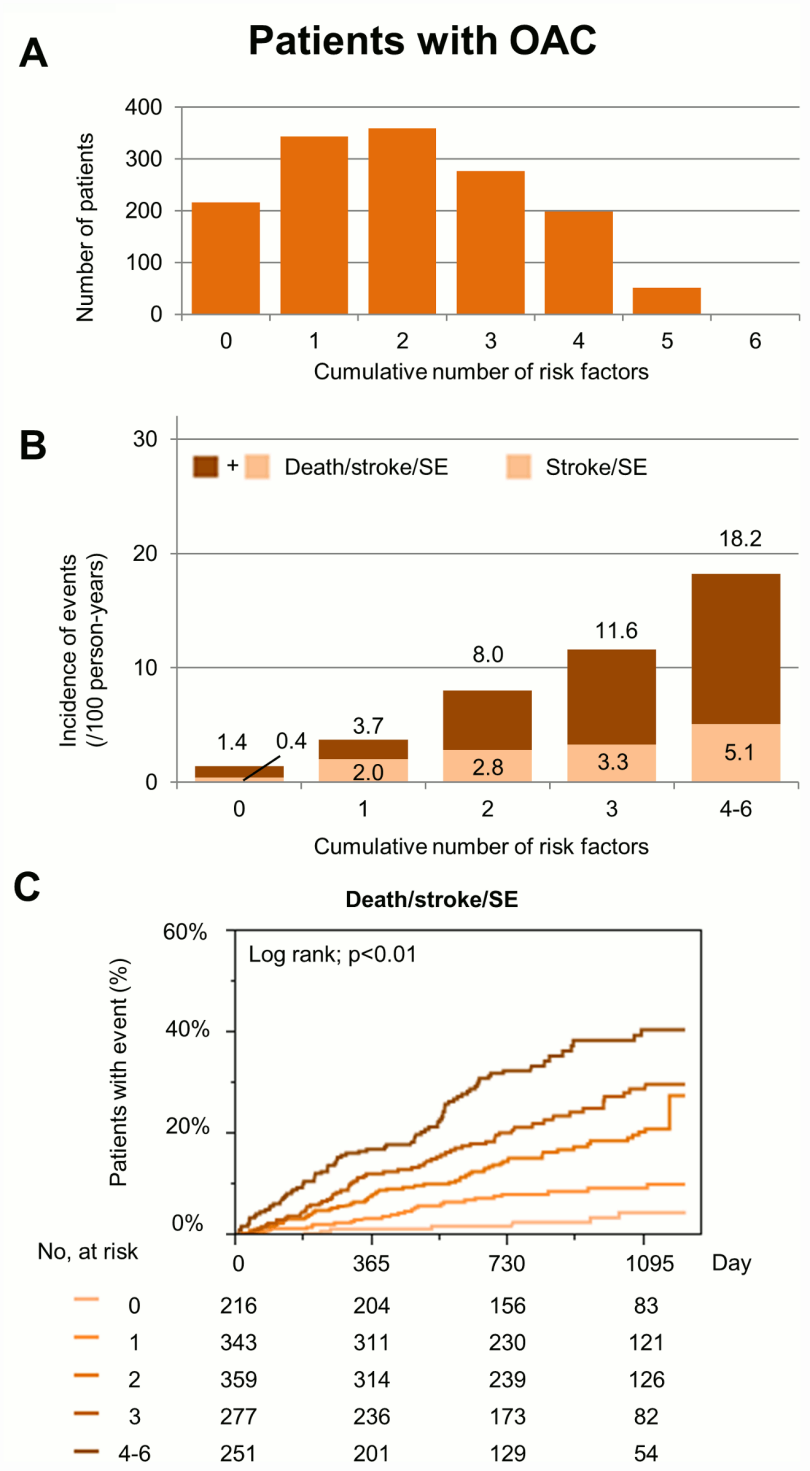
(A) The number of patients for each cumulative number of risk factors in patients with oral anticoagulant (OAC). (B) Incidence of death/stroke/systemic embolism (SE) during follow-up for each cumulative number of risk factors in patients with OAC. (C) Kaplan-Meier curves for the incidence of events during follow-up in patients with OAC. Risk factors are the following 6 components; advanced age, underweight, previous stroke/SE/TIA, heart failure, CKD, and anemia.

The numbers of patients and incidences of events for each cumulative number of 6 risk factors in patients taking OAC are shown in Fig 3, showing that cumulative number of 6 risk factors could also stratify the incidences of death/stroke/SE and stroke/SE in patients with OAC, as well as those without OAC.

Model predictive ability of the cumulative number of risk factors on the composite endpoint of ‘death/stroke/SE’ was good, with a C-index of 0.76 in patients without OAC, and 0.70 in patients taking OAC, respectively.

## Discussion

In this large community-based prospective survey, we have shown that amongst Asian patients with AF, advanced age, underweight, previous stroke/SE/TIA, heart failure, CKD, and anemia were independently associated with the incidence of death/stroke/SE. There was good prediction for endpoint of death/stroke/SE using these 6 variables in patients without OAC, as well as those with OAC. This Japanese study represents one of the largest prospective cohorts of Asian AF patients.

We defined the primary endpoint as composite of death/stroke/SE in the present study. In our cohort of Japanese AF patients, the rate of death was 3 to 4 times higher than the rate of stroke/SE, and the rate of non-cardiac death was higher than previous reports [11, 12]. Some of the ‘non-cardiac deaths’ could be due to undiagnosed stroke, given the nature of registry studies. Also, the RCTs clearly show that anticoagulation (ie. the therapeutic intervention) significantly reduces stroke/systemic embolism (by 64%) and all-cause mortality (by 26%) [13], compared to control/placebo—thus, justifying use of this composite endpoint.

### CHADS_2_/CHA_2_DS_2_-VASc scores and death/stroke/SE

In our registry, congestive heart failure, advanced age (≥75 years), and history of stroke/SE/TIA were independent predictors for the incidence of death/stroke/SE. Meanwhile, hypertension, diabetes mellitus, or female sex were not significantly associated with increased risk of death/stroke/SE. They were reported to be risk factors for stroke, but the association has been inconsistent [14, 15]. Well-controlled hypertension and diabetes mellitus may not be a risk factor for adverse events of AF [16]. Moreover, neither hypertension nor diabetes mellitus was associated with the stroke in Chinese AF patients [17], and neither was female sex in Japanese patients [18], suggesting that the risk factors for stroke/SE may possibly differ between Western and Asian countries.

### Anemia and death/stroke/SE in AF patients

Anemia is associated with increased mortality and morbidity in various cardiovascular diseases [19–21]. Anemia is also an independent predictor of 1-year all-cause death and re-hospitalization in elderly patients hospitalized with AF [22], and has been associated with major adverse cardiac and cerebrovascular events in patients with AF undergoing percutaneous coronary intervention [23]. A plausible mechanism whereby anemia increases the mortality may be a high risk of coronary ischemic events [19, 20], but cardiac death was relatively small in our registry. Alternatively, the higher rate of all-cause death and cardiovascular events might be related to the higher risk profile in anemic patients and underlying diseases causing anemia. Despite some studies of increased mortality and morbidity in anemic patients with cardiovascular diseases, the results of therapeutic intervention trials targeting anemia have been inconsistent [23]. Although we cannot address whether anemia is a mediator and therefore potentially amenable to therapeutic intervention, or merely a marker of adverse events in AF patients, our data showed that anemia is an independent, and strongest predictor for death/stroke/SE on multivariate analysis.

### CKD and death/stroke/SE in AF patients

CKD is considered to be an independent predictor for stroke/SE in AF [24–26], although it does not incrementally add to existing stroke risk scores [27]. CKD is also considered to be an independent predictor for composite endpoints of death/stroke/SE [28, 29]. Multiple possible mechanisms exist for the association between CKD and increased risks of death and cardiovascular diseases; reduced kidney function is associated with increased levels of inflammatory factors, enhanced coagulability, arterial calcification, arterial stiffness, and endothelial dysfunction [30]. Our data show that CKD was independently associated with increased risk of death/stroke/SE in AF patients, consistent with previous studies.

### Underweight and death/stroke/SE in AF patients

Being overweight has been associated with a lower risk of all-cause mortality and cardiovascular mortality in AF [31–33], while another study suggested sex-related differences [34]. These studies excluded underweight patients with a BMI <18.5 kg/m^2^, and the characteristics of underweight patients with AF are unknown. Japanese patients with AF are generally small and lean, and the Fushimi AF Registry contains many frail elderly patients who are underweight. We recently demonstrated that underweight was associated with a higher risk of stroke/SE, despite that the mechanisms remain speculative [35]. Our study showed the association between underweight and death/stroke/SE, but the impact of promoting weight gain in patients with AF is controversial. The recent trial suggests that weight reduction is actually associated with better outcomes in AF [36].

### Study limitations

Our study has several limitations. First, our data are based on an observational study and provides only associative evidence, not causative. Second, OAC was prescribed to only 53% of all the participating patients, and 60% of the patients with CHADS_2_ score ≥2 points in our registry, based on the responsible attending physician. However, OAC is often underused amongst AF patients in daily clinical practice, due to concerns about the bleeding complications [37, 38]. Our registry included many old and frail patients, who were perhaps considered unsuitable for OAC. Third, we did not collect the data of time in therapeutic range in patients with warfarin, and we only stratified entire cohort by OAC prescription at baseline. Indeed, there were some changes in the status of OAC prescription seen during follow-up period. However, our sensitivity analysis still shows that the 6 risk factors remained independently associated with the risk of death/stroke/SE in patients who had never taken any OAC throughout the follow-up period. Whilst exclusion of any OAC use during follow-up may result in conditioning for the future, but despite this limitation, our risk stratification is still predictive of the composite endpoint. Fourth, among 1,541 patients without OAC, 233 patients’ BMI data and 111 patients’ laboratory data of hemoglobin were missing, leading to selection bias. Fifth, the number of patients and incidence of endpoints might be underpowered to detect the impact of some risk factors, such as hypertension and diabetes mellitus. Finally, all enrolled patients were Japanese, thus the body compositions or other factors might differ from other populations. Our risk factors require external validation, with a view to development of risk stratification schemes that can be used in Asian AF patients.

## Conclusions

In one of the largest ‘real-world’ non-anticoagulated prospective cohorts of Asian AF patients, we show that advanced age, underweight, previous stroke/SE/TIA, heart failure, CKD, and anemia were independently associated with the incidence of death/stroke/SE. Such risk factors may lead to development of risk stratification schemes for use in Asian AF patients.
